# Supplementary material for: Epitope profiling using computational structural modelling demonstrated on coronavirus-binding antibodies
Source: PLoS Comput Biol. 2021 Dec 13;17(12):e1009675. doi: 10.1371/journal.pcbi.1009675 (PMC8700021; doi:10.1371/journal.pcbi.1009675)
Supplement: S1 Table — Antibodies with a solved crystal structures in complex with a SARS-CoV-2 antigen. Antibodies were assigned to their nearest epitope cluster, as defined by Dejnirattisai et al. [28]. Antibodies whose epitope cluster is labelled with a star (*) were not included in the previous analysis by Dejnirattisai et al. [28]. The new cluster label ‘Shoulder’ indicates that antibody’s epitope lies between the left and right shoulder clusters. (PDF) [file pcbi.1009675.s002.pdf]

Antibody	PDB Code	Citation	Epitope Cluster
B38	7BZ5	[1]	Neck
Bamlanivimab (LY-CoV555)	7KMG	[2]	*Left Shoulder
BD-236	7CHB	[3]	Neck
BD-368-2	7CHC	[3]	Left Shoulder
BD-604	7CHF	[3]	Neck
BD-629	7CH5	[3]	Neck
C102	7K8M	[4]	*Neck
C1A-B3	7KFW	[5]	*Neck
C1A-B12	7KFV	[5]	*Neck
C1A-C2	7KFX	[5]	*Neck
C1A-F10	7KFY	[5]	*Neck
CB6	7C01	[6]	Neck
CC12.1	6XC2	[7]	Neck
CC12.3	6XC4	[7]	Neck
COVA1-16	7JMW	[8, 9]	Left Flank
COVA2-04	7JMO	[8, 10]	Neck
COVA2-39	7JMP	[8, 10]	Right Shoulder
COVOX-45	7BEL	[11]	Right Flank
COVOX-75	7BEO	[11]	Right Shoulder
COVOX-88	7BEL	[11]	Left Shoulder
COVOX-150	7BEI	[11]	Neck
COVOX-158	7BEJ	[11]	Neck
COVOX-253	7BEN	[11]	Left Shoulder
COVOX-253H55L	7BEO	[11]	Left Shoulder
COVOX-269	7BEM	[11]	Neck
COVOX-316	7BEH	[11]	Left Shoulder
COVOX-384	7BEP	[11]	Left Shoulder
CR3022	7JN5	[12, 13]	Left Flank
CV07-250	6XKQ	[14]	Right Shoulder
CV07-270	6XKP	[14]	Left Shoulder
CV30	6XE1	[15, 16]	Neck
EY6A	6ZER	[17]	Left Flank
Fab_15033-7	7KLH	[18]	*Right Shoulder
Fab-52	7K9Z	[19]	*Left Shoulder
Fab-298	7K9Z	[19]	*Right Shoulder
Fab2-15	7L5B	[20, 21]	*Shoulder
LY-CoV481	7KMI	[2]	Neck
LY-CoV488	7KMH	[2]	Neck
MW06	7DPM	[22]	*Left Flank
P4A1	7CJF	[23]	Neck
P2B-2F6	7BWJ	[24]	Left Shoulder
P2C-1F11	7CDI	[25]	*Neck
P2C-1A3	7CDJ	[26]	*Shoulder
Regdanvimab	7CM4	[27]	*Right Shoulder
S2H14	7JX3	[28]	Right Shoulder
S304	7L0N	[29, 28]	Left Flank
S309	7JX3	[29, 28]	Right Flank
STE90-C11	7B30	[30]	*Neck

S1 Table. SARS-CoV-2 binding antibodies. Antibodies with a solved crystal structures in complex with a SARS-CoV-2 antigen. Antibodies were assigned to their nearest epitope cluster, as defined by Dejnirattisai *et al.* [11]. Antibodies whose epitope cluster is labelled with a star (*) were not included in the previous analysis by Dejnirattisai *et al.* [11]. The new cluster label 'Shoulder' indicates that antibody's epitope lies between the left and right shoulder clusters.

Bibliography

1. Wu Y, Wang F, Shen C, Peng W, Li D, et al. A noncompeting pair of human neutralizing antibodies block COVID-19 virus binding to its receptor ACE2. Science. 2020;368(6496):1274–1278. doi:10.1126/science.abc2241.

2. Jones BE, Brown-Augsburger PL, Corbett KS, Westerndorf K, Davies J, et al. The neutralizing antibody, LY-CoV555, protects against SARS-CoV-2 infection in non-human primates. Sci Transl Med. 2021; p. eabf1906. doi:10.1126/scitranslmed.abf1906.

3. Du S, Cao Y, Zhu Q, Wang G, Du X, et al. Structures of potent and convergent neutralizing antibodies bound to the SARS-CoV-2 spike unveil a unique epitope responsible for exceptional potency. bioRxiv. 2020;doi:10.1101/2020.07.09.195263.

4. Robbiani DF, Gaebler C, Muecksch F, Lorenzi JC, Wang Z, et al. Convergent antibody responses to SARS-CoV-2 in convalescent individuals. Nature. 2020;584(7821):437–442. doi:10.1038/s41586-020-2456-9.

5. Clark SA, Clark LE, Pan J, Coscia A, McKay LG, et al. Molecular basis for a germline-biased neutralizing antibody response to SARS-CoV-2. bioRxiv. 2020;doi:10.1101/2020.11.13.381533.

6. Shi R, Shan C, Duan X, Chen Z, Liu P, et al. A human neutralizing antibody targets the receptor-binding site of SARS-CoV-2. Nature. 2020;584(7819):120–124. doi:10.1038/s41586-020-2381-y.

7. Rogers TF, Zhao F, Huang D, Beutler N, Burns A, et al. Isolation of potent SARS-CoV-2 neutralizing antibodies and protection from disease in a small animal model. Science. 2020;369(6506):956–963. doi:10.1126/science.abc7520.

8. Brouwer PJ, Caniels TG, van der Straten K, Snitselaar JL, Aldon Y, et al. Potent neutralizing antibodies from COVID-19 patients define multiple targets of vulnerability. Science. 2020;369(6504):643–650. doi:10.1126/science.abc5902.

9. Liu H, Wu NC, Yuan M, Bangaru S, Torres JL, et al. Cross-neutralization of a SARS-CoV-2 antibody to a functionally conserved site is mediated by avidity. Immunity. 2020;53(6):1272–1280. doi:10.1016/j.immuni.2020.10.023.

10. Wu NC, Yuan M, Liu H, Lee CCD, Zhu X, Bangaru S, et al. An alternative binding mode of IGHV3-53 antibodies to the SARS-CoV-2 receptor binding domain. Cell Rep. 2020;33(3):108274. doi:10.1016/j.celrep.2020.108274.

11. Dejnirattisai W, Zhou D, Ginn HM, Duyvesteyn HM, Supasa P, et al. The antigenic anatomy of SARS-CoV-2 receptor binding domain. Cell. 2021;doi:10.1016/j.cell.2021.02.032.

12. Ter Meulen J, Van Den Brink EN, Poon LL, Marissen WE, Leung CS, et al. Human monoclonal antibody combination against SARS coronavirus: synergy and coverage of escape mutants. PLoS Med. 2006;3(7):e237. doi:10.1371/journal.pmed.0030237.

13. Wu NC, Yuan M, Bangaru S, Huang D, Zhu X, et al. A natural mutation between SARS-CoV-2 and SARS-CoV determines neutralization by a cross-reactive antibody. PLoS Pathog. 2020;16(12):e1009089. doi:10.1371/journal.ppat.1009089.

14. Kreye J, Reincke SM, Kornau HC, Sánchez-Sendin E, Corman VM, et al. A SARS-CoV-2 neutralizing antibody protects from lung pathology in a COVID-19 hamster model. Cell. 2020;183(4):1058–1069. doi:10.1016/j.cell.2020.09.049.

15. Seydoux E, Homad LJ, MacCamy AJ, Parks KR, Hurlburt NK, et al. Characterization of neutralizing antibodies from a SARS-CoV-2 infected individual. bioRxiv. 2020;doi:10.1101/2020.05.12.091298.

16. Hurlburt NK, Seydoux E, Wan YH, Edara VV, Stuart AB, et al. Structural basis for potent neutralization of SARS-CoV-2 and role of antibody affinity maturation. Nat Commun. 2020;11(1):5413. doi:10.1038/s41467-020-19231-9.

17. Zhou D, Duyvesteyn HM, Chen CP, Huang CG, Chen TH, et al. Structural basis for the neutralization of SARS-CoV-2 by an antibody from a convalescent patient. Nat Struct Mol Biol. 2020;27(10):950–958. doi:10.1038/s41594-020-0480-y.

18. Miersch S, Li Z, Saberianfar R, Ustav M, Case JB, et al. Tetravalent SARS-CoV-2 Neutralizing Antibodies Show Enhanced Potency and Resistance to Escape Mutations. bioRxiv. 2020;doi:10.1101/2020.10.31.362848.

19. Rujas E, Kucharska I, Tan YZ, Benlekbir S, Cui H, et al. Multivalency transforms SARS-CoV-2 antibodies into broad and ultrapotent neutralizers. bioRxiv. 2020;doi:10.1101/2020.10.15.341636.

20. Liu L, Wang P, Nair MS, Yu J, Rapp M, et al. Potent neutralizing antibodies against multiple epitopes on SARS-CoV-2 spike. Nature. 2020;584(7821):450–456. doi:10.1038/s41586-020-2571-7.

21. Rapp M, Guo Y, Reddem ER, Liu L, Wang P, et al. Modular basis for potent SARS-CoV-2 neutralization by a prevalent VH1-2-derived antibody class. Cell Rep. 2021;35(1):108950. doi:10.1016/j.celrep.2021.108950.

22. Wang J, Jiao S, Wang R, Zhang J, Zhang M, et al. Crystal structure of SARS-CoV-2 Spike RBD in complex with MW06 Fab. 2020;doi:10.2210/pdb7DPM/pdb.

23. Guo Y, Huang L, Zhang G, Yao Y, Zhou H, Shen S, et al. A SARS-CoV-2 neutralizing antibody with exceptional spike binding coverage and optimized therapeutic potentials. Research Square (Nature Preprint). 2020;doi:10.21203/rs.3.rs-78945/v1.

24. Ju B, Zhang Q, Ge J, Wang R, Sun J, et al. Human neutralizing antibodies elicited by SARS-CoV-2 infection. Nature. 2020;584(7819):115–119. doi:10.1038/s41586-020-2380-z.

25. Wang X, Zhang L, Ge J, Wang R. Crystal structure of SARS-CoV-2 antibody P2C-1F11 and RBD. 2020;doi:10.2210/pdb7CDI/pdb.

26. Wang X, Ge J. Crystal structure of SARS-CoV-2 antibody P2C-1A3 and RBD. 2020;doi:10.2210/pdb7CDJ/pdb.

27. Kim C, Ryu DK, Lee J, Kim YI, Seo JM, et al. A therapeutic neutralizing antibody targeting receptor binding domain of SARS-CoV-2 spike protein. Nat Commun. 2021;12(1):288. doi:10.1038/s41467-020-20602-5.

28. Piccoli L, Park YJ, Tortorici MA, Czudnochowski N, Walls AC, et al. Mapping neutralizing and immunodominant sites on the SARS-CoV-2 spike receptor-binding domain by structure-guided high-resolution serology. Cell. 2020;183(4):1024–1042. doi:10.1016/j.cell.2020.09.037.

29. Pinto D, Park YJ, Beltramello M, Walls AC, Tortorici MA, et al. Cross-neutralization of SARS-CoV-2 by a human monoclonal SARS-CoV antibody. Nature. 2020;583(7815):290–295. doi:10.1038/s41586-020-2349-y.

30. Bertoglio F, Fühner V, Ruschig M, Heine PA, Rand U, et al. A SARS-CoV-2 neutralizing antibody selected from COVID-19 patients by phage display is binding to the ACE2-RBD interface and is tolerant to known RBD mutations. bioRxiv. 2020;doi:10.1101/2020.12.03.409318.
