# Supplementary material for: Epitope profiling using computational structural modelling demonstrated on coronavirus-binding antibodies
Source: PLoS Comput Biol. 2021 Dec 13;17(12):e1009675. doi: 10.1371/journal.pcbi.1009675 (PMC8700021; doi:10.1371/journal.pcbi.1009675)
Supplement: S2 Table — Nanobodies with a solved crystal structures in complex with a SARS-CoV-2 antigen. Nanobodies were assigned to their nearest epitope cluster, as defined by Dejnirattisai et al. [28]. Nanobodies whose epitope cluster is labelled with a star (*) were not included in the previous analysis by Dejnirattisai et al. [28]. The new cluster label ‘Shoulder’ indicates that antibody’s epitope lies between the left and right shoulder clusters. (PDF) [file pcbi.1009675.s003.pdf]

Nanobody	PDB Code	Citation	Epitope Cluster
H11-D4	6YZ5	[1]	*Left Shoulder
H11-H4	6ZBP	[1]	*Left Shoulder
MR17	7C8W	[2, 3]	*Shoulder
MR17_K99Y	7CAN	[2]	*Shoulder
Nb20	7JVB	[4]	*Shoulder
SR4	7C8V	[2]	*Left Shoulder
SR31	7D2Z	[2]	*Left Shoulder
VHH-72	6WAQ	[5]	*Left Flank
VHH_E	7KN5	[6]	Left Shoulder
VHH_U	7KN5	[6]	*Left Flank
VHH_V	7KN6	[6]	*Left Flank
VHH_W	7KN7	[6]	*Left Flank

S2 Table. SARS-CoV-2 binding Nanobodies. Nanobodies with a solved crystal structures in complex with a SARS-CoV-2 antigen. Nanobodies were assigned to their nearest epitope cluster, as defined by Dejnirattisai *et al.* [7]. Nanobodies whose epitope cluster is labelled with a star (*) were not included in the previous analysis by Dejnirattisai *et al.* [7]. The new cluster label 'Shoulder' indicates that antibody's epitope lies between the left and right shoulder clusters.

References

1. Huo J, Le Bas A, Ruza RR, Duyvesteyn HM, Mikolajek H, et al. Neutralizing nanobodies bind SARS-CoV-2 spike RBD and block interaction with ACE2. Nat Struct Mol Biol. 2020;27(9):846–854. doi:10.1038/s41594-020-0469-6.

2. Li T, Cai H, Yao H, Zhou B, Zhao Y, et al. A potent synthetic nanobody targets RBD and protects mice from SARS-CoV-2 infection. BioRxiv. 2020;doi:10.1101/2020.06.09.143438.

3. Yao H, Cai H, Li T, Zhou B, Qin W, et al. A high-affinity RBD-targeting nanobody improves fusion partner's potency against SARS-CoV-2. PLoS Pathog. 2021;17(3):e1009328. doi:10.1371/journal.ppat.1009328.

4. Xiang Y, Nambulli S, Xiao Z, Liu H, Sang Z, et al. Versatile and multivalent nanobodies efficiently neutralize SARS-CoV-2. Science. 2020;370(6523):1479–1484. doi:10.1126/science.abe4747.

5. Wrapp D, De Vlieger D, Corbett KS, Torres GM, Wang N, et al. Structural basis for potent neutralization of betacoronaviruses by single-domain camelid antibodies. Cell. 2020;181(5):1004–1015. doi:10.1016/j.cell.2020.04.031.

6. Koenig PA, Das H, Liu H, Kümmerer BM, Gohr FN, et al. Structure-guided multivalent nanobodies block SARS-CoV-2 infection and suppress mutational escape. Science. 2021;371(6530):eabe6230. doi:10.1126/science.abe6230.

7. Dejnirattisai W, Zhou D, Ginn HM, Duyvesteyn HM, Supasa P, et al. The antigenic anatomy of SARS-CoV-2 receptor binding domain. Cell. 2021;doi:10.1016/j.cell.2021.02.032.
