# Supplementary material for: Epitope profiling using computational structural modelling demonstrated on coronavirus-binding antibodies
Source: PLoS Comput Biol. 2021 Dec 13;17(12):e1009675. doi: 10.1371/journal.pcbi.1009675 (PMC8700021; doi:10.1371/journal.pcbi.1009675)
Supplement: S3 Table — Analysis of structural epitope overlap between epitope group members. Epitope residues were defined as those within 4.5A of the antibody in the crystal structure. Analysis was conducted using Arpeggio [62]. (PDF) [file pcbi.1009675.s004.pdf]

| Epitope Group | No. of Group Members | Average No. of Epitope Residues (min, max) | Number Of Unique Epitope Residues Across Group | Min Residue Overlap Between Any Two Group Members | Max Residue Overlap Between Any Two Group Members | Average Residue Overlap Between Any Two Group Members | No. of Residues Present In The Epitope of All Group Members | No. of Residues Present In The Epitope of 90%+ of Group Members | No. of Residues Present In The Epitope of 50%+ of Group Members |
|---|---|---|---|---|---|---|---|---|---|
| Neck | 23 | 31.9 (19, 38) | 49 | 7 | 34 | 27.2 | 5 | 23 | 32 |
| Left Shoulder | 10 | 20.3 (13, 26) | 69 | 1 | 19 | 7.9 | 0 | 1 | 16 |
| Left Flank | 5 | 25.4 (22, 28) | 62 | 4 | 23 | 10.8 | 3 | 3 | 22 |
| Right Flank | 2 | 22.5 (21, 24) | 39 | 6 | 6 | 6 | 6 | 6 | 39 |
| Right Shoulder | 6 | 21.8 (13, 27) | 56 | 2 | 18 | 9.7 | 1 | 1 | 20 |
| Shoulder | 2 | 23.0 (21, 25) | 29 | 17 | 17 | 17 | 17 | 17 | 17 |

**S3 Table. SARS-CoV-2 binding antibodies epitope interactions.** Analysis of structural epitope overlap between epitope group members. Epitope residues were defined as those within 4.5A of the antibody in the crystal structure. Analysis was conducted using Arpeggio [1].

# References


1. Jubb HC, Higueruelo AP, Ochoa-Montaño B, Pitt WR, Ascher DB, et al. Arpeggio: a web server for calculating and visualising interatomic interactions in protein structures. J Mol Biol. 2017;429(3):365–371. doi:10.1016/j.jmb.2016.12.004.
