# Supplementary material for: Epitope profiling using computational structural modelling demonstrated on coronavirus-binding antibodies
Source: PLoS Comput Biol. 2021 Dec 13;17(12):e1009675. doi: 10.1371/journal.pcbi.1009675 (PMC8700021; doi:10.1371/journal.pcbi.1009675)
Supplement: S4 Table — Analysis of the paratope-epitope interactions between antibody and SARS-CoV-2 RBD in the cocrystal structures, determined using Arpeggio [62]. (PDF) [file pcbi.1009675.s005.pdf]

Antibody	Van der Waals	Hydrogen Bond	Weak Hydrogen Bond	Ionic	Aromatic	Hydrophobic	Carbonyl	Polar	Weak Polar
B38	9	20	13	0	3	34	1	33	13
Bamlanivimab	5	11	3	7	10	59	1	10	3
BD-236	9	13	13	1	1	27	0	22	16
BD-368-2	5	14	13	0	5	26	1	25	16
BD-604	6	15	14	0	0	27	1	23	9
BD-629	5	13	9	0	7	25	1	23	15
C102	9	13	6	0	16	33	0	20	18
C1A-B3	9	16	14	1	4	26	0	26	12
C1A-B12	5	17	12	1	4	36	1	23	14
C1A-C2	13	22	15	1	8	36	1	29	14
C1A-F10	11	17	11	1	6	33	1	23	16
CB6	11	15	10	2	7	38	0	19	14
CC12.1	19	22	18	2	7	38	0	30	26
CC12.3	7	11	15	0	8	33	0	18	13
COVA1-16	9	8	8	0	0	23	1	12	17
COVA2-04	10	14	14	1	4	31	0	20	12
COVA2-39	8	7	8	0	10	48	0	13	18
COVOX-150	11	21	12	4	5	39	2	33	17
COVOX-158	10	16	15	0	5	19	0	21	13
COVOX-253	3	8	5	0	8	36	1	9	4
COVOX-253H55L	8	7	6	0	11	39	1	9	6
COVOX-269	17	20	13	2	6	36	2	30	17
COVOX-316	4	4	3	0	11	52	0	11	5
COVOX-384	4	7	3	4	3	38	0	9	5
COVOX-45	6	16	11	8	5	39	1	20	19
COVOX-75	13	9	9	0	1	36	0	11	10
COVOX-88	10	9	1	4	10	37	0	12	13
CR3022	9	13	13	10	0	35	0	14	11
CV07-250	3	14	9	0	11	34	0	16	9
CV07-270	9	12	5	9	6	37	1	15	9
CV30	10	14	11	0	0	23	0	22	11
EY6A	11	7	8	1	3	34	1	11	15
Fab_15033-7	8	10	9	4	11	36	0	13	15
Fab-298	1	6	2	0	1	20	0	10	6
Fab-52	6	3	5	2	2	34	0	5	10
Fab2-15	6	4	5	0	10	27	0	6	16
LY-CoV481	14	13	15	0	2	21	0	21	13
LY-CoV488	8	19	9	3	3	22	1	29	15
MW06	7	9	15	0	3	17	4	14	18
P2B-2F6	4	4	3	2	2	16	0	7	8
P2C-1A3	16	8	8	0	13	52	0	11	15
P2C-1F11	8	11	11	0	2	27	0	21	14
P4A1	16	20	13	1	4	33	1	28	16
Regdanvimab	9	8	5	7	15	57	0	11	3
S2H14	8	13	13	0	0	2	0	17	17
S304	9	10	12	0	3	36	1	16	12
S309	6	12	7	2	0	43	1	14	14
STE90-C11	5	14	10	1	7	38	0	22	14
Average	*8.5*	*12.3*	*9.6*	*1.7*	*5.5*	*33.1*	*0.5*	*17.9*	*12.8*

S4 Table. SARS-CoV-2 binding antibodies paratope interactions. Analysis of the paratope-epitope interactions between antibody and SARS-CoV-2 RBD in the cocrystal structures, determined using Arpeggio [1].

References

1. Jubb HC, Higueruelo AP, Ochoa-Montaño B, Pitt WR, Ascher DB, et al. Arpeggio: a web server for calculating and visualising interatomic interactions in protein structures. J Mol Biol. 2017;429(3):365–371. doi:10.1016/j.jmb.2016.12.004.
