# Supplementary material for: Epitope profiling using computational structural modelling demonstrated on coronavirus-binding antibodies
Source: PLoS Comput Biol. 2021 Dec 13;17(12):e1009675. doi: 10.1371/journal.pcbi.1009675 (PMC8700021; doi:10.1371/journal.pcbi.1009675)
Supplement: Attachment — Submitted filename: Response_to_reviewers.pdf [file pcbi.1009675.s006.pdf]

Dear Professor Fraternali,

We thank the editor and reviewers for their thorough feedback on our manuscript. We have responded fully to all comments raised in a point- by-point fashion below. All changes to the manuscript have been described and are marked in red in an attached PDF.

Reviewer 1 Comments

This paper by Robinson and colleagues explores structural-based clonal clustering. They show that taking into account structural properties reveals insights not apparent at the sequence level, which is both consistent with increasing bodies of work in the area, but also intriguing. While they focus mostly on COVID targeting antibodies, this should more be seen as a powerful dataset to provide insights that are much more broadly applicable.

We thank the reviewer for their kind words and for highlighting the broad applicability of our method. We agree that the method could be used for any disease-focussed antibody dataset of sufficient size and quality. We have changed the wording of our title, abstract and introduction to better describe the wider applicability of our method.

>TITLE

Old: Epitope profiling of coronavirus-binding antibodies using computational structural modelling

New: Epitope profiling using computational structural modelling demonstrated on coronavirus – binding antibodies

>ABSTRACT

Old: We describe a novel computational method for epitope profiling based on structural modelling and clustering. The method identifies sequence-dissimilar but functionally-similar antibodies across the Coronavirus Antibody Database, and achieves accuracy (92% of antibodies in multiple-occupancy structural clusters bind to consistent domains).

New: We describe a novel computational method for epitope profiling based on structural modelling and clustering. Using the method, we demonstrate that sequence dissimilar but functionally similar antibodies can be found across the Coronavirus Antibody Database, with high accuracy (92% of antibodies in multiple-occupancy structural clusters bind to consistent domains

>INTRODUCTION

New: We chose to apply our method to CoV-AbDab to illustrate the value of structural clustering on an example dataset. Our method could be applied to any high-quality disease-focussed antibody dataset to extract additional information and supplement existing clonotyping analyses.

The work is well written and will be of broad interest. My two main comments relate to validation and replication.

Validation - The authors illustrate the potential power of structure based clustering using spike rbd antibodies, but to reflect the broad title it should be validated across other antibody sets.

We thank the reviewer for raising this point. Our analysis of crystal structures focussed on spike-RBD antibodies due to crystal structure availability. At the time of the analysis, there were no published crystal structures of antibodies binding to non-RBD sites on SARS-CoV-2. On the other hand, our structural modelling and clustering analysis was applied to a general set of coronavirus-binding antibody sequences, including antibodies to different viruses [MERS-CoV, SARS-CoV-1 and SARS-CoV-2], antigens (Spike [S] and Nucleocapsid [N]), and antigen regions (including spike S1 non-RBD and spike S2). Therefore, whilst our analysis is focused on one disease, in this instance this corresponds to multiple viruses and multiple proteins, with many potential epitopes on each protein. Due to high global interest, the Coronavirus Antibody Database (CoV-AbDab) is dominated by antibodies targeting SARS-CoV-2 and specifically the spike RBD, often engaged by virus-neutralising antibodies of therapeutic interest. However, several of our clusters bin together antibodies targeting non-RBD regions (see SI file 1).

To validate our structural modelling and clustering pipeline in a new disease context would require either (a) hundreds of solved crystal structures against pathogen family-specific antigens or (b) hundreds of pathogen family-specific, epitope-labelled, Fv-domain antibody sequences. We are unaware of any other pathogen class that currently benefits from enough of either data beyond coronaviruses (e.g. for Ebola the most recent epitope-labelled antibody dataset is by Rijal *et al*. 2019; they report just 8 antibodies with epitope labels (largely non-overlapping) and do not supply the full Fv sequences). Indeed, this was our motivation for creating CoV-AbDab, to demonstrate the value that comes from compiling data on pathogen-specific antibodies into a single location. It is our hope that similar datasets for other pathogen families will soon be created, enabling us to validate our method in different disease contexts. We have added a section to the manuscript discussing this data deficit.

>DISCUSSION
New: We hope to create similar datasets for other pathogen families and suspect others will do the same, enabling the validation of computational structural profiling in multiple different disease contexts.

Replication - I would encourage the authors to make their scripts available to enable replication of the work (e.g. reference a github repository rather than just saying that internal scripts were used). This will also facilitate others to apply similar approaches to other datasets.

We agree with the reviewer and are happy to make our script available. The code for this project (Structural Profiling of Antibodies to Cluster by Epitope, "SPACE") is dependent on the entire SAbDab-SAbPred codebase, so we will shortly be providing it packaged as a Singularity container ('SAbBox-Singularity Version'), free for academic use. We have added a ‘Code and Data Availability’ section to the manuscript.

The following manuscript changes were made:

>CODE AND DATA AVAILABILITY
New: The properties of the clusters generated in this work are available in the SI, all model structures are freely available on Zenodo (doi: 10.5281/zenodo.5569157). The SPACE code is available through our SAbBox suite.

>METHODS - STRUCTURAL CLUSTERING ALGORITHM
New: We developed the Structural Profiling of Antibodies to Cluster by Epitope (SPACE) algorithm to structurally cluster the 1,500 models.

>DISCUSSION
New: Inspired by the structural similarity of antibodies that bind to the same epitope, we predicted and clustered the structures of the broad set of antibodies documented in CoV-AbDab using our SPACE algorithm

Reviewer 2 Comments

Robinson et al. describe a novel computational method for epitope profiling applied to SARS-CoV2 antibodies using structural clustering coupled with homology modelling, demonstrating a strong structural-functional relationship (that sequence-based clustering would fail to pick up). The work tackles a very relevant and topical problem, particularly in a pandemic context, and deserves publication.

We thank the reviewer for their kind words.

Are there any impacts on recent AlphaFold2/RosettaFold developments for this work that are worth commenting (or pursuing further analyses)? I understand this study predates the release of these two methods, but it might be a good opportunity to comment on implications at least.

We thank the reviewer for raising these recent developments in protein modelling. Improvements in modelling techniques may lead to improvements in the speed and accuracy of antibody structure prediction, which would likely improve structural clustering. We have added a sentence to the discussion section of the paper commenting on these developments.

The following manuscript changes were made:

>DISCUSSION
New: Additionally, given recent developments in ab initio single-domain protein modelling techniques (72), we may soon see significant improvements in the speed and accuracy of antibody structure prediction, and by extension antibody structure-based epitope profiling. Many alternative clustering methods exist beyond the template-based approach reported here, and these may be more appropriate to use when clustering models generated by future high-throughput structure modelling algorithms.

Lines 280-284. The statement around interaction analysis is limited to hydrogen bonds and a bit vague ("almost always"). Perhaps it would be interesting to quantify conservation of different interaction types, rather than just Hydrogen Bonds (could also provide a figure highlighting them).

We agree with the reviewer that further analysis of interaction types would be interesting. We have conducted further analysis and altered the wording accordingly. We have also produced a table of all of the interaction types between the antibody and epitope, using Arpeggio, which is available in the Supplementary Materials.

>DISCUSSION

Old: Using Arpeggio (58) to identify the type of binding interactions these FWRL3 residues were involved in, 9 paratopes contained hydrogen bonds between L83 and the RBD, almost always to RBD residue 498.

New: Using Arpeggio (58) to identify the type of binding interactions these FWRL3 residues were involved in, 9 paratopes contained hydrogen bonds between L83 and the RBD. These included residue 498 of the RBD in 8/9 cases. Most interactions between the antibody paratope and RBD epitope are hydrophobic (with an average of 33.1 per molecule), polar (average 17.9) and hydrogen bonds (average 12.3). For full Arpeggio analysis of interactions between antibodies and the RBD in the cocrystal complexes see Table S4.

>SI TABLES

	Number of interactions (by type) with SARS-CoV-2-RBD antigen in co-crystal structure								
Antibody Name	Van der Waals	Hydrogen_Bond	Weak_Hydrogen_Bond	Ionic	Aromatic	Hydrophobic	Carbonyl	Polar	Weak_Polar
LY-CoV488	8	19	9	3	3	22	1	29	15
P2B-2F6	4	4	3	2	2	16	0	7	8
COVOX-253H55L	8	7	6	0	11	39	1	9	6
P2C-1A3	16	8	8	0	13	52	0	11	15
BD-604	6	15	14	0	0	27	1	23	9
Bamlanivimab	5	11	3	7	10	59	1	10	3
LY-CoV481	14	13	15	0	2	21	0	21	13
Regdanvimab	9	8	5	7	15	57	0	11	3
COVOX-75	13	9	9	0	1	36	0	11	10
CB6	11	15	10	2	7	38	0	19	14
CC12.3	7	11	15	0	8	33	0	18	13
S309	6	12	7	2	0	43	1	14	14
STE90-C11	5	14	10	1	7	38	0	22	14
COVOX-269	17	20	13	2	6	36	2	30	17
CV30	10	14	11	0	0	23	0	22	11
COVOX-158	10	16	15	0	5	19	0	21	13
COVA1-16	9	8	8	0	0	23	1	12	17
COVA2-39	8	7	8	0	10	48	0	13	18
CR3022	9	13	13	10	0	35	0	14	11
C1A-B12	5	17	12	1	4	36	1	23	14
C1A-C2	13	22	15	1	8	36	1	29	14
CC12.1	19	22	18	2	7	38	0	30	26
B38	9	20	13	0	3	34	1	33	13
C1A-F10	11	17	11	1	6	33	1	23	16
C1A-B3	9	16	14	1	4	26	0	26	12
BD-236	9	13	13	1	1	27	0	22	16
P2C-1F11	8	11	11	0	2	27	0	21	14
BD-629	5	13	9	0	7	25	1	23	15
BD-368-2	5	14	13	0	5	26	1	25	16
EY6A	11	7	8	1	3	34	1	11	15
COVOX-316	4	4	3	0	11	52	0	11	5
COVOX-150	11	21	12	4	5	39	2	33	17
COVOX-253	3	8	5	0	8	36	1	9	4
C102	9	13	6	0	16	33	0	20	18
Fab-298	1	6	2	0	1	20	0	10	6
COVA2-04	10	14	14	1	4	31	0	20	12
CV07-270	9	12	5	9	6	37	1	15	9
S2H14	8	13	13	0	0	2	0	17	17
Fab-52	6	3	5	2	2	34	0	5	10
CV07-250	3	14	9	0	11	34	0	16	9
COVOX-384	4	7	3	4	3	38	0	9	5
COVOX-88	10	9	1	4	10	37	0	12	13
P4A1	16	20	13	1	4	33	1	28	16
Fab2-15	6	4	5	0	10	27	0	6	16
MW06	7	9	15	0	3	17	4	14	18
Fab_15033-7	8	10	9	4	11	36	0	13	15
S304	9	10	12	0	3	36	1	16	12
COVOX-45	6	16	11	8	5	39	1	20	19
Average	*8.5*	*12.3*	*9.6*	*1.7*	*5.5*	*33.1*	*0.5*	*17.9*	*12.8*

Table S4. Analysis of the paratope-epitope interactions between antibody and SARS-CoV-2 RBD in the cocrystal structures, determined using Arpeggio.

It would be interesting for the community to make the homology models generated available.
We agree with the reviewer. The models have been made available and a 'data and code availability' section has been added to the methods section.

Was there any sort of energy minimisation performed on the models?
Please clarify.

We thank the reviewer for the opportunity to clarify this. We did not perform energy minimisation on the models – this was deliberate as we have found through previous work that perturbing the coordinates inherited from the homology modelling templates reduces the average accuracy of ABodyBuilder (Leem *et al.* 2016). We want our models to be of the highest possible quality to ensure meaningful clustering.

Was there any sort of quality control performed on the models?
Please clarify.

We thank the reviewer for the opportunity to clarify this. Quality control is built into our ABodyBuilder modelling software, i.e. homology templates are not only chosen by Ramachandran suitability for the target CDR sequence, but also based on framework template graftability and through steric clash checks to the rest of the model. ABodyBuilder will not produce an entirely homology-modelled output if the model is inherently low-quality. In our work, we use only the 72.7% of antibodies that passed all these checks.

Have the authors tested different clustering approaches? (or what's the rationale behind choosing the one used) How would that affect their conclusions?

We chose a simple greedy clustering algorithm based on pre-computed template distance matrices because Department Talks and Events next week it is both rapid (important as the size of antigen-specific antibody databases continues to grow) and yields >90% domain-consistency. This methodology is limited in the fact that it relies on every CDR loop backbone being homology modellable, however in our use case this amounted to over 72% of the antibodies. We believe most clustering methods would give very similar results to our chosen algorithm, given the observed data distribution. We have added a sentence to the methods section explaining our choice of algorithm, and a comment to the discussion section regarding when other computational methods might be appropriate.

The following manuscript changes were made:

>METHODS
New: Greedy clustering was selected due to its simplicity, good performance, and speed, ensuring scalability across larger datasets.

>DISCUSSION
New: Additionally, given recent developments in ab initio single-domain protein modelling techniques (72), we may soon see significant improvements in the speed and accuracy of antibody structure prediction, and by extension antibody structure-based epitope profiling. Many alternative clustering methods exist beyond the template-based approach reported here, and these may be more appropriate to use when clustering models generated by future high-throughput structure modelling algorithms.

Minor Points:

Typo in the abstract: confidence -> confidence
Figure 3 (specially panel b) and 4 look low-res
Line 209 - There is no panel c in Figure 3. I am guessing it is a typo (Figure 2C instead?)
Line 372 - AUC (Area under curve? - please define abbreviation)

We thank the reviewer for spotting these. We have changed the typo in the abstract, remade figures 3 and 4 with increased resolution, changed the reference to Figure 3C to Figure 2C, and defined AUC as the area under the curve.

Reviewer 3 Comments

1. Arguably the IGHV3-53/IGHV3-66 example (line 205) is really an argument against the excessive stringency of the “identical heavy V gene” requirement, given that this particular pair of germline genes have near-identical sequences (the IMGT sequences for the *01 alleles of these V genes differ by only a single residue out of 97). The later example (line 489) of IGHV3-7 vs. IGHV3-30 (12 residue differences) is potentially more compelling.

We thank the reviewer for raising this point. We agree that the IGHV3-7 vs. IGHV3-30 example found through the structural clustering analysis is a good case of structural similarity across differing germlines. We have added a sentence to the structural clustering analysis commenting on the sequence differs between these antibodies.

The following manuscript changes were made:

>RESULTS

Old: In most cases where multiple clonotypes are found in the same structural cluster, it is due to significant differences in the CDRH3 sequence. However, some clusters such as SC134 (which pools COV2-2490 (60) with H712061+K711727 (61)), align closest to different heavy V (IGHV3-7 *vs*. IGHV3-30) and light V (IGKV1-5 *vs*. IGKV1D-16) genes.

New: In most cases where multiple clonotypes are found in the same structural cluster, it is due to significant differences in the CDRH3 sequence. However, some clusters such as SC134 (which pools COV2-2490 (60) with H712061+K711727 (61)), contain many differences across the entirety of the sequence (26 differences across VH, 27 across VL) and align closest to different heavy V (IGHV3-7 *vs*. IGHV3-30) and light V (IGKV1-5 *vs*. IGKV1D-16) genes.

2. There might be several ways to define “same” epitope (line 6 and others). It would be good to have some insight into how loose the working definition is here, e.g. by quoting the overlap of the structural epitopes derived from the structures of bound antibodies considered to bind to the same epitope.

We agree with the reviewer that there can be many definitions of epitope. We follow the epitope groups as set out in Dejnirattisai et al. 2021. We have also conducted further analysis of the epitopes using Arpeggio (Jubb *et al*. 2017), which we have added the full details of to the Supplementary Material (see table below). The overlap in structural epitopes (defined as residues within 4.5A of the antibody in the crystal structure) varies by epitope group. For the most well-defined group of antibodies, binding the neck region, over 90% of the antibodies bind to the same 32 epitope residues. The average overlap in epitope between any two members of the group is 27 residues out of 32 total residues in the epitope (85%, on average). The other epitope groups are more diverse, with only 1 residue found consistently in 9/10 of the left shoulder antibody epitopes, and 1 residue in 6/6 of the right shoulder antibody epitopes. However, both groups have epitope residues shared across at least half of their members, at 16 and 20 residues respectively.

The following manuscript changes were made:

>RESULTS

Epitope Binning.
For a full analysis of structural epitope overlap between antibodies within epitope groups, see Table S3.

>SI TABLES

New:

Epitope Group	Number of Group Members	Average Number of Epitope Residues (min, max)	Number of Unique Epitope Residues Across Group	Minimum Number of Residues Overlap Between Any Two Group Members	Maximum Number of Residues Overlap Between Any Two Group Members	Average Number of Residues Overlap Between Any Two Group Members (As A % Of Average Epitope Size)	Number of Residues Present In The Epitope of All Group Members	Number of Residues Present In The Epitope of >=90% of Group Members	Number of Residues Present In The Epitope of >=50% of Group Members
Neck	23	31.9 (19, 38)	49	7	34	27.2 (85.2%)	5	23	32
Left Shoulder	10	20.3 (13, 26)	69	1	19	7.9 (38.9%)	0	1	16
Left Flank	5	25.4 (22, 28)	62	4	23	10.8 (42.5%)	3	3	22
Right Flank	2	22.5 (21, 24)	39	6	6	6 (26.7%)	6	-	-
Right Shoulder	6	21.8 (13, 27)	56	2	18	9.7 (44.5%)	1	1	20
Shoulder	2	23.0 (21, 25)	29	17	17	17 (73.9%)	17	-	-

Table S3: Analysis of structural epitope overlap between epitope group members. Epitope residues were defined as those within 4.5A of the antibody in the crystal structure. All analysis conducted using Arpeggio.

3. There is no direct discussion of epitope constraints — some topologies, or the presence of glycans, may constrain the viable positions and orientations of an antibody more than others, making structural (and sequence) similarity more likely. Presumably some evolutionarily-conserved epitope topologies may be associated with tighter constraints than others, and that is likely to have an impact on the associated antibody structural clusters. This consideration appears relevant to the discussion about conservation and vulnerability (from line 411), and should be addressed.

We thank the reviewer for raising this and we agree that certain epitope constraints will impact structural and sequence similarity of binding antibodies. We have added a section to the discussion section to comment on this.

The following manuscript changes were made:

>RESULTS

New: An open question remains as to how strictly an antibody's structure needs to be conserved to engage the same epitope. This is likely to be highly epitope dependent. For example, for epitopes naturally suited to VH-dominated engagement, less selection pressure would act upon light chain structure and vice versa, while some epitope topologies and environments (e.g. extent of glycosylation) may also exert different levels of pressure on complementary antibody geometries.

Two minor points:

The abbreviation RBD is defined after its first usage in line 119.

There is a typo: confidenfce

We have removed the second RBD definition and changed the typo in the abstract.
